# Molecules, Morphometrics and Modeling of the Medically Important Genus *Hemiscorpius* Peters, 1861 (Scorpiones: Hemiscorpiidae) in Iran Reveal New Species from Kerman

**DOI:** 10.3390/insects17010018

**Published:** 2025-12-23

**Authors:** Hossein Dehghan, Esmail Amiri Ghanat Saman, Seyed Massoud Madjdzadeh, Masoumeh Amiri, Asma Moeinadini, Lorenzo Prendini, Hossein Barahoei

**Affiliations:** 1 Department of Public Health, School of Health, Jiroft University of Medical Sciences, Jiroft P.O. Box 7861634200, Iran; h.dehghan31@yahoo.com; 2 Bio Environmental Health Hazards Research Center, Jiroft University of Medical Sciences, Jiroft P.O. Box 7861634200, Iran; 3 Department of Biology, Faculty of Sciences, Shahid Bahonar University of Kerman, Kerman P.O. Box 7616913439, Iran; madjdzadeh@uk.ac.ir (S.M.M.); moeinadini999@gmail.com (A.M.); 4 Department of Biology, Faculty of Science, Lorestan University, Khorramabad P.O. Box 6815144316, Iran; amirsam927@yahoo.com; 5 Arachnology Lab and Scorpion Systematics Research Group, Division of Invertebrate Zoology, American Museum of Natural History, Central Park West at 79th Street, New York, NY 10024-5192, USA; lorenzo@amnh.org; 6 Agriculture Institute, Research Institute of Zabol, Zabol P.O. Box 9861673831, Iran

**Keywords:** *Hemiscorpius*, morphology, morphometry, phylogeny, species distribution modeling, Iran

## Abstract

Eight species of the medically important scorpion genus *Hemiscorpius* Peters, 1861 have been reported in Iran, three of which are responsible for most of the severe clinical cases of envenomation. However, morphological similarity complicates species delimitation in this genus, hindering the identification of species implicated in envenomations. The present study integrates morphology, DNA sequences, and ecological niche modeling to clarify the taxonomy and distribution of *Hemiscorpius* in southern Iran, providing taxonomic insights relevant to public health and biodiversity conservation. Morphometric analyses were performed to evaluate size and shape differences; molecular phylogenetic analyses were conducted on DNA sequences of the mitochondrial Cytochrome *c* Oxidase Subunit I gene; and species distribution models, based on occurrence records and bioclimatic variables, were developed. Morphometric analyses revealed significant interspecific differences and sexual dimorphism. A new species was identified and described as *Hemiscorpius aratta* sp. n. Molecular phylogenetic analysis confirmed the distinctiveness of the new species and revealed intraspecific variation in the type species, *Hemiscorpius lepturus* Peters, 1861, suggesting possible cryptic diversity. Southern Iran, particularly the coastline of the Persian Gulf and the Gulf of Oman, represents a diversity hotspot for *Hemiscorpius*. Topographical barriers such as the Jebal Barez, Makkoran, and Zagros Mountain ranges promoted isolation and speciation, leading to high levels of endemism in the genus. Ecological niche models revealed that the distributions of *Hemiscorpius* species are strongly influenced by temperature and precipitation. Coastal species are restricted to thermally stable maritime habitats, whereas semi-arid species occupy regions with higher temperature seasonality. Range-restricted species are habitat specialists, vulnerable to environmental change. This study reinforces the importance of integrating morphological, molecular, and ecological data for resolving taxonomic ambiguity.

## Simple Summary

Eight species of the dangerous scorpion genus *Hemiscorpius* have been identified in Iran, three of which are responsible for most of the severe cases of envenomation. Due to their marked morphological similarity, accurately distinguishing between these species is difficult. In the present study, researchers examined the morphological traits, DNA sequences, and habitat models of *Hemiscorpius* species in southern Iran. The results revealed clear differences among the species and led to the discovery of a new one named *Hemiscorpius aratta* sp. n. Southern Iran, especially the coasts of the Persian Gulf and the Gulf of Oman, was identified as a hotspot for these scorpions. Mountain ranges such as the Zagros, Makkoran, and Jebal Barez have contributed to the isolation and formation of new species. Temperature and rainfall were found to be the main environmental factors influencing their distribution. Species with specialized habitat requirements are more vulnerable to environmental changes.

## 1. Introduction

The family Hemiscorpiidae Pocock, 1893 comprises a single genus, *Hemiscorpius* Peters, 1861, with 20 species, distributed from northeast Africa, across the Arabian Peninsula, through Iraq and Iran, to Pakistan [1,2]. Species of *Hemiscorpius* are lithophilous [3] inhabitants of arid to semi-arid habitats from flat coastal areas to piedmont and mountains, regions that exhibit considerable climatic variation [4,5,6,7].

Eight species of the genus have been reported in Iran [6,7,8]: *Hemiscorpius acanthocercus* Monod & Lourenço, 2005; *Hemiscorpius enischnochela* Monod & Lourenço, 2005; *Hemiscorpius gaillardi* (Vachon, 1974); *Hemiscorpius jiroftensis* Amiri Ghanat Saman et al., 2025; *Hemiscorpius kashkayi* Karataş & Moradi-Gharkheloo, 2013; *Hemiscorpius lepturus* Peters, 1861; *Hemiscorpius persicus* (Birula, 1903); *Hemiscorpius shahii* Kovařík et al., 2017. At least three of these species are considered medically important, their envenomations responsible for morbidity and mortality in humans [6].

Iran is among the countries most affected by scorpionism, with approximately 40,000 to 50,000 cases of envenomation and around 20 deaths reported annually [9,10]. Epidemiological studies indicate that 10–15% of envenomations in southwest Iran are caused by *H. lepturus*, the species responsible for 89% of fatalities [11]. Published reports highlight serious clinical symptoms associated with envenomation by *H. acanthocercus*, *H. enischnochela*, and *H. lepturus*, including hemoglobinuria, proteinuria, hematuria, hemolysis of blood cells, and increased creatinine excretion [12,13,14]. No reports have implicated other Iranian species of *Hemiscorpius*.

Considering the medical importance of *Hemiscorpius*, a robust taxonomy, with a reliable means of species identification, is essential. However, the taxonomy of *Hemiscorpius* is complicated by the marked morphological similarity among species, hindering accurate identification of the species implicated in envenomations. Although several studies have investigated the systematics of Iranian *Hemiscorpius* species from morphological and molecular perspectives [6,7,15,16,17,18,19], an integrative approach has yet to be applied to the systematics of the entire genus.

The present study integrates morphology, DNA sequences, and ecological niche modeling to clarify the taxonomy and distribution of *Hemiscorpius* in southern Iran, providing taxonomic insights relevant to public health and biodiversity conservation. Morphometric analyses were performed to evaluate size and shape differences; molecular phylogenetic analyses were conducted on DNA sequences of the mitochondrial Cytochrome *c* Oxidase Subunit I (COI) gene; and species distribution models, based on occurrence records and bioclimatic variables, were developed. Morphometric analyses revealed interspecific variation and sexual dimorphism. A new species was identified and described as *Hemiscorpius aratta* sp. n. Molecular phylogenetic analysis confirmed the distinctiveness of the new species and revealed intraspecific variation in the type species, *Hemiscorpius lepturus* Peters, 1861, suggesting possible cryptic diversity.

Southern Iran, particularly the coastline of the Persian Gulf and the Gulf of Oman, represents a diversity hotspot for *Hemiscorpius*. Topographical barriers such as the Jebal Barez, Makkoran, and Zagros Mountain ranges promoted isolation and speciation, leading to high levels of endemism in the genus. Ecological niche models revealed that the distributions of *Hemiscorpius* species are strongly influenced by temperature and precipitation. Coastal species are restricted to thermally stable maritime habitats whereas semi-arid species occupy regions with higher temperature seasonality. Range-restricted species are habitat specialists, vulnerable to environmental change. The study reinforces the importance of integrating morphological, molecular, and ecological data for resolving taxonomic ambiguity in medically important genera.

## 2. Materials and Methods

### 2.1. Fieldwork

Field surveys were conducted across southern Iran, encompassing the provinces of Fars, Hormozgan, Kerman, Khuzestan, and Sistan & Baluchistan, from 2019 to 2024. Sampling locations focused on areas with significant reports of scorpion stings and fatalities [6,14] and areas previously unsampled or undersampled for *Hemiscorpius*. Specimens were collected by searching potential shelters (e.g., under stones) by day and with ultraviolet lamps at night. All specimens were preserved in 80% ethanol and stored at room temperature. Geographical data were recorded using a Garmin GPSMAP^®^ 78s (Olathe, KS, USA) device.

### 2.2. Material and Morphology

Species were identified using the diagnostic key of Shahi & Barahoei [6]. Material examined is deposited in the Medical Entomology Collection of Jiroft University of Medical Sciences (JMU), Iran, the Research Institute of Zabol (RIZ), Iran, the Zoological Museum of Shahid Bahonar University of Kerman (ZMBK), Iran, and the American Museum of Natural History (AMNH), New York, including the Ambrose Monell Cryocollection (AMCC). Morphology was examined using a Labomed Luxeo 6Z stereomicroscope (Los Angeles, CA, USA). Morphological terminology follows Stahnke [20], Sissom [21], and Prendini [22] for general nomenclature, Loria & Prendini [23] for lateral ocelli, and González-Santillán & Prendini [24] for pedipalpal and metasomal carinae.

### 2.3. Morphometrics

Standard morphometrics, following Stahnke [20] and Sissom et al. [25], were recorded under the stereomicroscope using an Accud Digital Point Caliper (Suzhou, China). Twenty-three measurements (mm) and 11 morphometric ratios were recorded from 88 adult specimens, representing six species: *H. acanthocercus* (13♂, 10♀); *H*. *aratta* sp. n. (8♂, 7♀); *H. enischnochela* (16♂, 10♀); *H. jiroftensis* (6♂, 2♀); *H. lepturus* (7♂, 4♀); *H. persicus* (2♂, 3♀). Univariate and multivariate statistical analyses follow Barahoei et al. [26,27,28] and Amiri et al. [29]. A Shapiro-Wilk test was conducted to confirm that variables met assumptions of normality. Morphological variation among populations was examined by evaluating two aspects of body form, i.e., size and shape. Size (hereafter “overall size”), calculated as the square root of the sum of all the traits, is a reasonable estimate of body surface area [30,31]. The log-shape ratios method, in which each of the measured traits was divided by the overall size of each individual and its logarithm calculated, was used to remove the effect of size from the variables [31,32]. Shape data were size-corrected, because size may be influenced by the environment and by developmental conditions [33]. Type II two-way ANOVA and MANOVA were performed according to species and sex factors to evaluate whether populations differed in size or shape. When ANOVA revealed a significant difference in size, a post hoc Tukey Honestly Significant Difference (HSD) test was performed for pairwise comparison among species separately for species with sexual dimorphism or nondimorphism. Box plots were constructed to visualize interspecific differences in overall size. Statistical analyses of morphometric data were conducted using the following packages in R 2 [34]. The “MASS” package [35] was used for linear discriminant analysis (LDA), the “car” package [36] for ANOVA II, the “Agricolae” package3 for the Tukey HSD test, and the “Ggplot2” package [37] for the box plots. Phenotypic differences between populations were analyzed with LDA using the population factor [38].

### 2.4. Molecular Analyses

DNA was extracted from pedipalp muscle tissue (stored in 96% ethanol and kept in a refrigerator) using the FavorPrep Tissue Genomic DNA Extraction Mini Kit (FAVORGEN, Pingtung, Taiwan). DNA quantity and concentration were determined using a DS-11 spectrophotometer (DeNovix, Wilmington, DE, USA). The mitochondrial COI gene was PCR amplified using primers LCO1490 and HCO2198 [39] and the thermocycler protocol described by Barahoei et al. [27]. PCR products were evaluated using agarose gel electrophoresis and high-quality samples sent to Niagen Noor Company (Tehran, Iran) for purification and sequencing. Only the forward primer was used for sequencing. Sequence quality was checked, and sequences edited and aligned using BioEdit 7.0.5.3, with default parameters [40]. No partitioning by codon position was applied due to the limited size of the dataset. The molecular dataset comprised 32 sequences for six Iranian species of *Hemiscorpius*, including 13 newly generated sequences (four each from *H. aratta* sp. n. and *H. lepturus*, two from *H. enischnochela*, and three from *H. persicus*), and 19 sequences from GenBank. Samples and sequences of three Iranian species, *H. gaillardi*, *H. kashkayi*, and *H. shahii*, were unavailable. *Scorpio palmatus* (Ehrenberg, 1828) was used as the outgroup. Genetic divergence within and between species and the phylogenetic information content of the sequences were evaluated using MEGA 11 [41]. The best-fitting nucleotide substitution model (TIM3 + G) was selected using jModelTest2 on the CIPRES website [42]. Bayesian inference (BI) was performed for 50,000,000 generations with 5,000 discarded as burnin, using MrBayes 3.2.7a [43] on the CIPRES website [42]. Phylogenetic trees were visualized and edited using FigTree 1.4.04 and Adobe Photoshop 26.8.1.

### 2.5. Ecological Niche Modeling

In the present study, 138 occurrence records were obtained for eight Iranian species of *Hemiscorpius*: 39 for *H. acanthocercus*; 34 for *H. enischnochela*; 29 for *H. lepturus*; 11 for *H. jiroftensis*; seven for *H. aratta* sp. n.; and six each for *H. kashkayi*, *H. persicus*, and *H. shahii*. *Hemiscorpius gaillardi*, represented by only a single point locality, was excluded from the modeling due to insufficient data. Ecological niche models (ENMs) were developed using MaxEnt 3.3.3e [44] and OpenModeller 1.0.7 [45], with spatial data processed in ArcMap 10 [46]. Nineteen bioclimatic variables from WorldClim 2.1 [47], along with a derived slope layer, were used in the modeling process. Environmental layers were clipped to Iran’s boundaries using ArcMap. Correlations between climatic and topographic variables were assessed in OpenModeller to avoid redundancy. Modeling was conducted for the years 1970–2000 to predict suitable habitats across southern and southwestern Iran. Model performance and variable contributions were evaluated to identify climatic and topographic factors influencing species distributions. Default settings were used for model calibration, with 5,000 iterations and the minimum training value averaged across 15 replicates as the default convergence threshold. Due to the four smaller sample sizes, the cross-validation option was used for model calibration and performance, and the results averaged to estimate species niches and distributions. Twenty-five percent of the occurrence localities were randomly withheld for evaluating the statistical significance of the model. The area under the curve (AUC), which summarizes the model’s ability to rank presence localities higher than a sample of random pixels [48], was calculated. AUC values of ≤0.5 correspond to predictions equal to or worse than random. AUC values of >0.5 are generally poor predictions (0.5 to 0.7), reasonable predictions (0.7 to 0.9), or very good predictions (>0.90); see Manel et al. [48] for caveats on the use of AUC with presence/background data. After running MaxEnt and obtaining continuous logistic output maps (0–1), habitat suitability was classified using the tenth percentile training presence logistic threshold. This threshold was applied to convert continuous predictions into suitable and unsuitable areas, minimizing the influence of potential outliers in the presence data and ensuring a conservative delineation of suitable habitat.

## 3. Results

### 3.1. Morphometric Analyses

The results of a type II two-way ANOVA for overall size (species × sex; F = 5.87; *p* < $10^{-2}$) were significant between species. Sexual dimorphism in size was significant (F = 178.82; *p* < $10^{-15}$). The *t*-test results for 23 morphometric characters and 11 ratios for *Hemiscorpius* species indicated that 24 characters were sexually dimorphic and ten characters were not (Table 1). Males were larger in 16 characters and females larger in eight. Four meristic characters (MT2L–MT5L) and four ratios (MT2L/W–MT5L/W) were significant in all species with higher numbers in males than females. The number of sexually dimorphic characters observed in *H. acanthocercus* was 21 (13 characters larger in males and eight in females), followed by *H. aratta* sp. n., with 18 (11 characters larger in males and seven in females); *H. lepturus*, with 17 (13 characters larger in males and four in females); *H. enischnochela*, with 12 (larger in males); *H. persicus*, with 10 (larger in males); and *H. jiroftensis*, with eight (larger in males).

ANOVA and Tukey HSD tests performed for characters without sexual dimorphism (Table S1) indicated that *H. enischnochela* and *H. jiroftensis* were larger than the other species for eight characters and *H. aratta* sp. n. was smaller for seven. Among sexually dimorphic characters in which males were larger (Table S2), *H. jiroftensis* was larger than other species and *H. acanthocercus* smaller for 14 characters each whereas, for two characters in which females were larger (Table S3), *H. acanthocercus* was smaller. 

**Figure 1:**
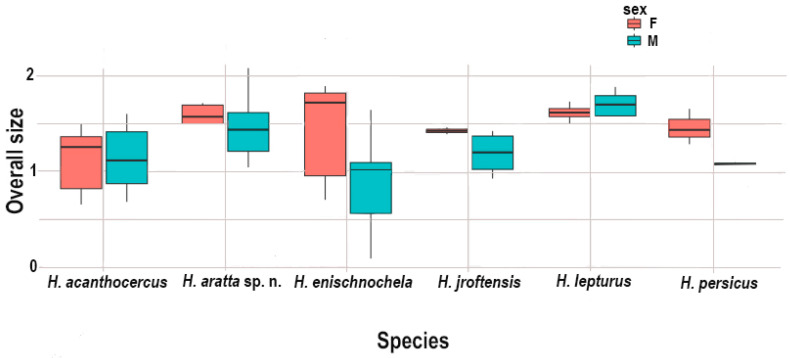
Box plots illustrating overall size of six Iranian species of *Hemiscorpius* Peters, 1861. Females indicated in red (left), males in blue (right).

The box plot for overall size (Figure 1) revealed that *H. enischnochela* is larger than the other species and *H. acanthocercus* smaller. The type II MANOVA performed for shape data revealed significant differences between species (F = 298.73; *p* < $10^{-15}$) and sexes (F = 60.00; *p* < $10^{-15}$) with a significant difference for their interaction (F = 2.11; *p* < 0.01). This suggests that shape may be under strong selection both among and within species. Selection reflects a direct relationship between female size, fertility, and egg production [25]. Furthermore, sexual dimorphism in shape is dissimilar among species. Therefore, the effect of sex was removed in subsequent analyses.

**Figure 2:**
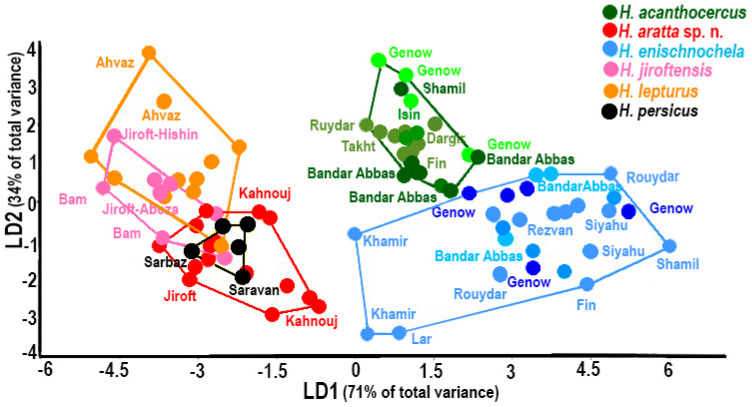
Linear discriminant analysis of six Iranian species of *Hemiscorpius* Peters, 1861 based on sex-corrected shape data.

In the LDA on the species factor using sex-corrected shape variables (Figure 2), the first two components (LD1 and LD2) explained 71% and 34% of the variance, respectively. In this analysis, changes in the first axis were based on pedipalp chela manus length and carapace width, and changes in the second axis, on pedipalp chela manus length and movable finger length. Although some species were sampled from common locations, the described species of *Hemiscorpius* formed well-defined phenetic groups. *Hemiscorpius acanthocercus* and *H. enischnochela* were situated close to each other and well separated from the other species along the first axis.

### 3.2. Molecular Analyses

**Figure 3:**
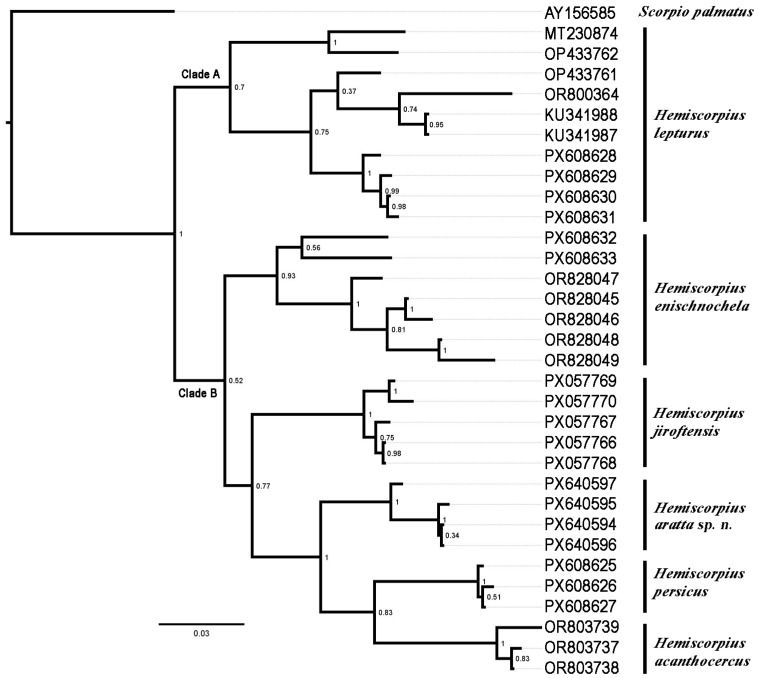
Phylogeny of six Iranian species of *Hemiscorpius* Peters, 1861 based on Bayesian inference of Cytochrome *c* Oxidase Subunit I sequences. *Scorpio palmatus* (Ehrenberg, 1828) is used as outgroup. Posterior probability values indicated at nodes. Sample numbers correspond to Table 2.

The final alignment of the COI sequences (Table 2) consisted of 627 bp, comprising 422 (67.3%) conserved sites, 205 (32.7%) variable sites, and 141 (22.5%) parsimony-informative sites. Genetic distances were 0.067 between the new species and *H. persicus*, 0.078 between the new species and *H. acanthocercus*, *H. enischnochela*, and *H. jiroftensis*, and 0.103 between the new species and *H. lepturus* (Table 3). In the tree obtained with BI (Figure 3), *H. lepturus* (Clade A) grouped sister to a clade comprising the other five species included in the analysis (Clade B), in which the species were arranged successively, as follows: (*H. enischnochela* (*H. jiroftensis* (*H. aratta* sp. n. (*H. acanthocercus* + *H. persicus*)))).

### 3.3. Ecological Niche Modeling

**Figure 4:**
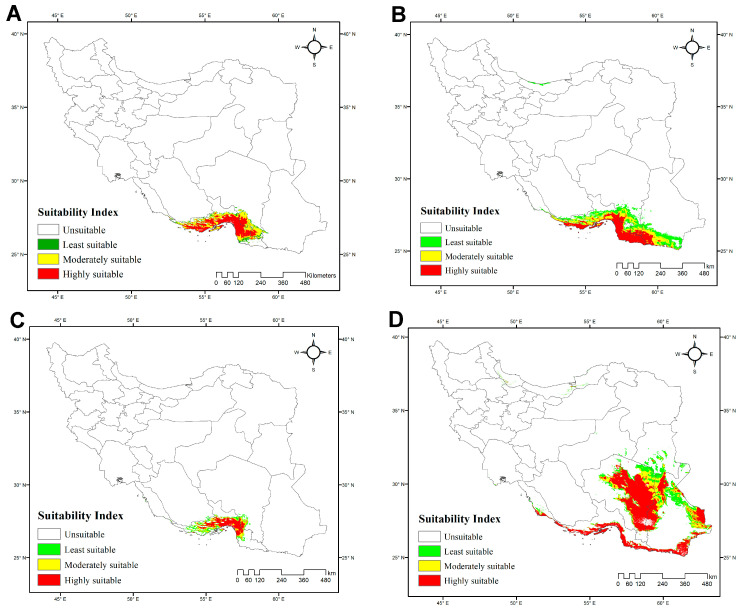
Potentially suitable habitats for four Iranian species of *Hemiscorpius* Peters, 1861 under current climatic conditions: (**A**) *Hemiscorpius acanthocercus* Monod & Lourenço, 2005; (**B**) *Hemiscorpius aratta* sp. n.; (**C**) *Hemiscorpius enischnochela* Monod & Lourenço, 2005; (**D**) *Hemiscorpius jiroftensis* Amiri Ghanat Saman et al., 2025.

The average AUC values for each species model suggest that the model predictions are accurate: *H. acanthocercus*, 0.984; *H. aratta* sp. n., 0.893; *H. enischnochela*, 0.986; *H. jiroftensis*, 0.948; *H. kashkayi*, 0.998; *H. lepturus*, 0.936; *H. persicus*, 0.965; *H. shahii*, 0.98. The highest habitat suitability for *H. acanthocercus* occurs in the coastal and near-coastal zones of Hormozgan Province, with moderate suitability extending into southern Kerman Province and western Sistan & Baluchistan Province. Peripheral transitional belts exhibited low suitability, and most of Iran was predicted as unsuitable (Figure 4A; Table S4). Among the environmental predictors, the minimum temperature of the coldest month (BIO6) was the most influential, followed by the mean diurnal range (BIO2) and temperature seasonality (BIO4) (Table 4), underscoring the importance of temperature-related constraints, particularly winter cold. The model estimated the total extent of suitable habitat at 55,299 km^2^ (3% of Iran’s land area), with unsuitable habitats covering 1,592,896 km^2^ (97%).

The habitat-suitability model for *H. aratta* sp. n. indicates a predominantly coastal distribution along the northern shores of the Persian Gulf and the Gulf of Oman. Unsuitable areas comprise 1,288,471 km^2^ (78%) of Iran, whereas suitable habitat totals 359,723 km^2^ (22%) (Figure 4B; Table S4). The model identifies BIO7 (temperature annual range) as the dominant predictor (Table 4), pointing to maximal suitability where the annual difference between the warmest- and coldest-month temperatures is relatively small—i.e., maritime belts and low-elevation forelands. This spatial signal is consistent with the definition of BIO7 and its known role as a proxy for continental versus maritime environments, with lower values typically occurring near the coastline.

The model for *H. enischnochela* revealed a strikingly restricted distribution along the northern coastline of the Persian Gulf and the Gulf of Oman, particularly in Hormozgan Province, with no suitable habitat elsewhere (Figure 4C; Table S4). Unsuitable areas encompass 1,595,335 km^2^ (97% of the country), whereas suitable habitats cover only 52,859 km^2^ (3%). The analysis identified the minimum temperature of the coldest month (BIO6) as the strongest predictor, followed by precipitation of the coldest quarter (BIO19) (Table 4). These variables suggest that the species’ distribution is constrained to maritime-influenced regions characterized by mild winters and the maintenance of minimum thermal thresholds, coupled with moderate levels of cold-season precipitation. Such areas correspond to low-elevation forelands and coastal belts of southern Iran, where seasonal extremes are buffered compared to the continental interior.

**Figure 5:**
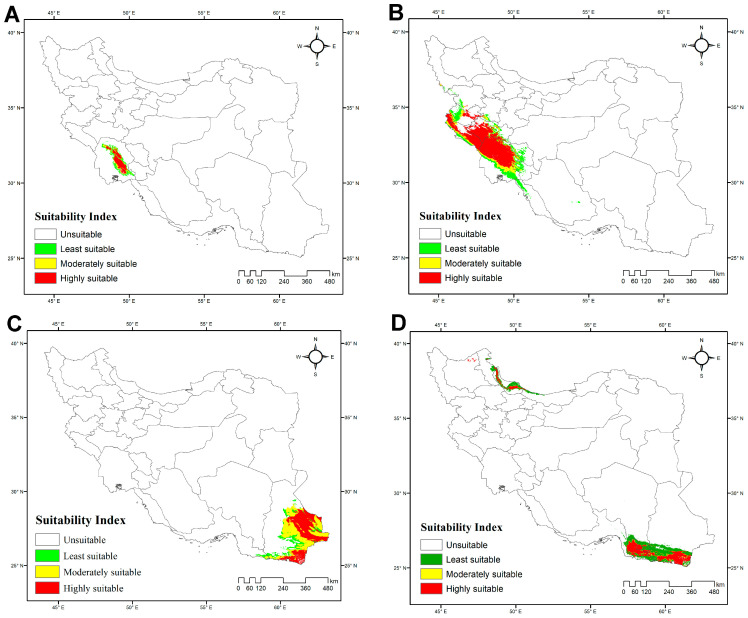
Potentially suitable habitats for four Iranian species of *Hemiscorpius* Peters, 1861 under current climatic conditions: (**A**) *Hemiscorpius kashkayi* Karataş & Moradi-Gharkheloo, 2013; (**B**) *Hemiscorpius lepturus* Peters, 1861; (**C**) *Hemiscorpius persicus* (Birula, 1903); (**D**) *Hemiscorpius shahii* Kovařík et al., 2017.

The model for *H. lepturus* indicates that highly suitable habitats are concentrated in southwestern Iran, particularly in Khuzestan and Ilam provinces. Moderately suitable areas form peripheral zones around the core, gradually extending into regions of lower suitability, whereas the least suitable and unsuitable areas dominate central and eastern Iran (Figure 5B; Table S4). Among environmental variables, precipitation of the coldest quarter (BIO19) emerged as the most influential factor, followed by precipitation of the driest month (BIO14) (Table 4). The total area of suitable habitats was estimated at 98,987 km^2^ (6% of Iran), whereas unsuitable habitats cover 1,549,207 km^2^ (94%).

The model for *H. persicus* revealed a geographically limited yet distinct pattern of habitat suitability across Iran. Suitable areas are concentrated in the eastern and southern parts of Sistan & Baluchistan Province, in southeastern Iran. Most of Iran is unsuitable, reflecting strong environmental constraints on the species’ potential range (Figure 5C; Table S4). Among the bioclimatic variables, isothermality (BIO3) had the greatest influence, followed by precipitation of the wettest quarter (BIO16) and mean temperature of the coldest quarter (BIO11) (Table 4). The total area of suitable habitats was estimated at 293,135 km^2^ (18% of Iran), whereas unsuitable habitats cover 1,355,059 km^2^ (82%).

The model for *H. jiroftensis* revealed that this species predominantly inhabits southeastern and southern Iran, particularly the provinces of Hormozgan, Kerman, and Sistan & Baluchistan (Figure 4D; Table S4). These areas encompass approximately 610,996 km^2^, accounting for about 37% of Iran’s land area. The remaining regions, including central, western, and northern Iran, covering approximately 1,037,197 km^2^ or 63% of the country, are unsuitable. Variable contribution analysis identified temperature seasonality (BIO4) as the most influential factor, followed by annual precipitation (BIO12). Precipitation of the warmest quarter (BIO18) also played a role in habitat suitability (Table 4).

The model for *H. kashkayi* indicates a highly localized and restricted habitat suitability pattern, concentrated in a narrow area of southwestern Iran, primarily in Khuzestan Province. Moderately and least suitable habitats occur in limited bands surrounding this core, whereas the rest of Iran is unsuitable (Figure 5A; Table S4). Maximum temperature of the warmest month (BIO5) was identified as the most influential factor shaping the distribution of this species (Table 4). The total area of suitable habitats was estimated at 14,664 km^2^, representing only 1% of Iran’s land area, whereas unsuitable habitats cover 1,633,529 km^2^ (99%).

The model for *H. shahii* identified two primary regions of Iran with varying suitability. The most suitable areas are concentrated along the southeastern coastal provinces bordering the Gulf of Oman, in Hormozgan and Sistan & Baluchistan provinces. Moderately suitable and least suitable zones surround these core areas, whereas the majority of the country’s interior is unsuitable (Figure 5D; Table S4). A small patch of highly suitable habitat along the southern Caspian Sea coast is an artifact of limited presence data. BIO4 (Temperature Seasonality) was found to be the most influential environmental predictor (Table 4). The total suitable area covers approximately 59,711 km^2^ (3.6% of Iran), whereas the unsuitable area extends over 1,588,484 km^2^ (96.4% of the country).

### 3.4. Systematics

Morphological examination of 164 specimens (100 males and 64 females) of *Hemiscorpius* from Iran resulted in the identification of six distinct species, including a new species, *Hemiscorpius aratta* sp. n., described below.

**Family Hemiscorpiidae Pocock, 1893**

**Genus *Hemiscorpius* Peters 1861**

***Hemiscorpius acanthocercus* Monod & Lourenço, 2005**

**Figure 6:**
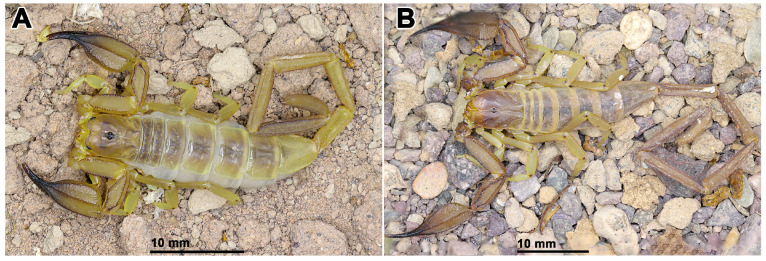
In vivo habitus of adult males of two Iranian species of *Hemiscorpius* Peters, 1861: (**A**) *Hemiscorpius aratta* sp. n.; (**B**) *Hemiscorpius jiroftensis* Amiri Ghanat Saman et al., 2025.

**Figure 7:**
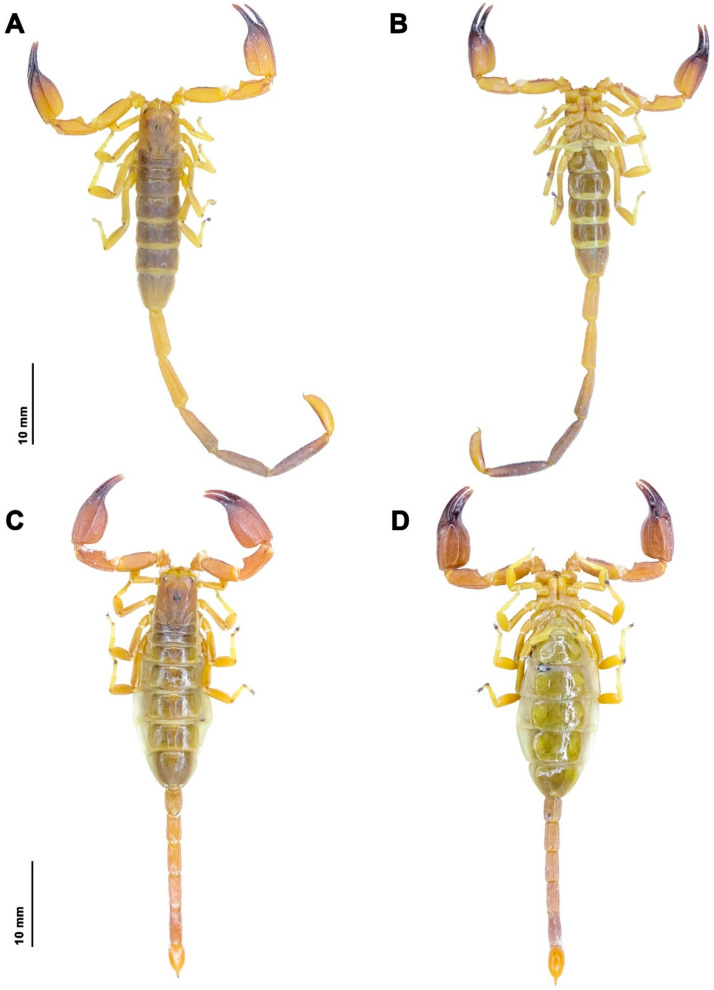
*Hemiscorpius aratta* sp. n., habitus, dorsal aspect (**A**,**C**) and ventral aspect (**B**,**D**): (**A**,**B**) Holotype male (RIZ [HD-30]); (**C**,**D**) Paratype female (RIZ [HD-28]).

**Figure 8:**
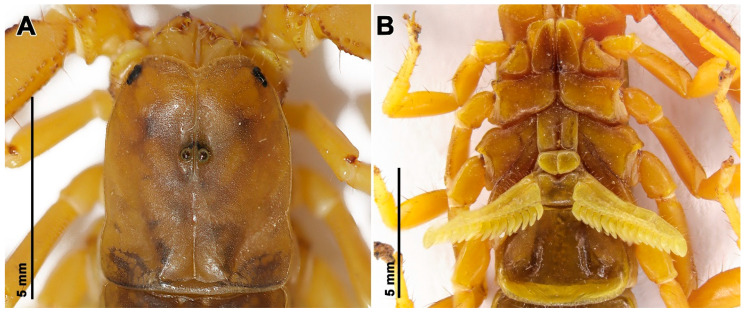
Hemiscorpius aratta sp. n., holotype male (RIZ HD-30): (**A**) Carapace; (**B**) Sternum and pectines.

*Hemiscorpius acanthocercus* Monod & Lourenço, 2005: 874–886, Figures 1C, D, 2–7, 36; Navidpour et al., 2013: 17, Figures 12, 24–27; Salari & Sampour, 2017: 102, 104, Figures 6–8 (in part); Shahi & Barahoei, 2023: 1592, 1593, Figures 2, 3.

**Material examined: IRAN:** *Hormozgan Prov.*: Bandar Abbas Co.: 27°14′57.4″ N 56°21′16.3″ E, 2013, M.H. Speed, 4♂, 4♀ (RIZ [Hem-84]), 30/IV/2021, M. Shahi, 2♂, 1♀ (RIZ [Hem-164]); Dargir, 27°19′50″ N 56°13′46.1″ E, 2014, M. Shahi, 1♂, 1♀ (RIZ [Hem-139]); Fin, 27°37′54.8″ N 55°54′22.4″ E, 2019, S. Aflaki, 2♂ (RIZ [Hem-78]); Genow village, 27°26′47″ N 56°18′20.2″ E, 2014, M. Shahi, 4♂, 3♀ (RIZ [Hem-156]); Isin, 27°22′54″ N 57°09′20.2″ E, 17/IX/2021, M. Shahi, 3♂, 1♀ (RIZ [Hem-97]); Shamil, 27°32′56.2″ N 56°50′10″ E, 30/XII/2012, M. Ghasemi, 4♂, 2♀ (RIZ [Hem-131]); Takht, 27°30′13.9″ N 56°37′50.6″ E, 22/IV/2011, O. Jafari, 2♂, 2♀ (RIZ [Hem-02]); Khamir Co.: Ruydar, 27°28′18.8″ N 55°25′38.1″ E, 2014, Sh. Jahanbani, 3♂, 3♀ (RIZ [Hem-117]); Rudan Co.: Abnama, 27°27′25.6″ N 57°15′48.3″E, 1/V/2013, R. Habibi, 1♂, 2♀ (RIZ [Hem-29]); Islamabad, 27°22′54″ N 57°09′20.2″ E, 25/V/2013, A. Ranjbari, 2♂, 1♀ (RIZ [Hem-130]).

**Distribution:** Endemic to Hormozgan and Kerman provinces in southern Iran [6].

***Hemiscorpius aratta* Barahoei & Prendini sp. n.**

*Hemiscorpius acanthocercus* Monod & Lourenço, 2005: Gorouhi et al., 2023: 326, Figures 2d, 5 [misidentification]; Adeli-Sardou et al., 2024: 647, Figures 2, 3 [misidentification].

urn:lsid:zoobank.org:act:48F7073E-4C8D-4F7E-8B90-447C40913489

Figures 1–3, 4B, 6A, 7–10, Table 1, Table 2, Table 3, Table 4 and Table 5

**Type Material.** Holotype ♂ (RIZ [HD-30]), **IRAN:** *Kerman Prov.*: Sahlavar, 27°51′52″ N 57°39′08″ E, 13/IV/2021, H. Dehghan. Paratypes: **IRAN:** *Kerman Prov.*: Kahnouj, 27°52′11″ N, 57°33′40″ E, 11/IV/2021, H. Dehghan & E. Amiri, 1♂ (RIZ [HD-31]), 2♀ (JMU [HD-33, HD-34]); Rameshk, 26°44′46″ N, 58°42′01″ E, 13/VII/2024, H. Dehghan, 1♂ (JMU [HD-37]); Sahlavar, 27°56′43″ N, 57°38′08″ E, 13/IV/2021, H. Dehghan, 1♂ (AMNH [HD-38]), 1♂ (JMU [HD-38]), 15/VI/2022, Ahmadzadeh, 2♂, 1♀ (JMU [HD-14]), 2♀ (JMU [HD-36]), 2♂, 1♀ (RIZ [HD-28]), Ghale Gang, Marz, 58°14′26″ N, 26°43′58″ E, 30/VIII/2023, 2♂ (JMU [HD-29, HD-32]); Marz, 58°14′23″ N, 26°43′15″ E, 20/X/2021, H. Dehghan, 1♀ (JMU [HD-35], 1♂ [HD-40]).

**Diagnosis.** *Hemiscorpius aratta* sp. n. (Figure 6A) is most closely related to *H. jiroftensis* (Figure 6B) from which it may be distinguished as follows. The new species is smaller, with total length less than 68.9 mm in the male and 48.4 mm in the female (Table 5), than *H. jiroftensis*, with total length 65.4–90.4 mm in the male and 60.1–69.2 mm in the female. The superciliary carinae of the carapace are smooth and three smooth surfaces are present near the lateral ocelli in the new species whereas the superciliary carinae are granular and only one smooth surface is present near the lateral ocelli in *H. jiroftensis*. The pedipalp chela movable finger is shorter than the chela manus in the new species (Table 5) but longer in *H. jiroftensis*. Metasomal segments III–V are darker than segments I and II in the male of the new species (Figure 7) whereas all segments are dark in the male of *H. jiroftensis* (Figure 6B). Tergite VII is wider than long in the male of the new species but longer than wide in the male of *H. jiroftensis*.

**Etymology.** The name is a noun in apposition taken from Aratta, a semi-mythical civilization from Jiroft and surrounding regions of Iran, mentioned in Sumerian literature, and known for its wealth, skilled artisans, and cultural influence on early Mesopotamia.

**Figure 9:**
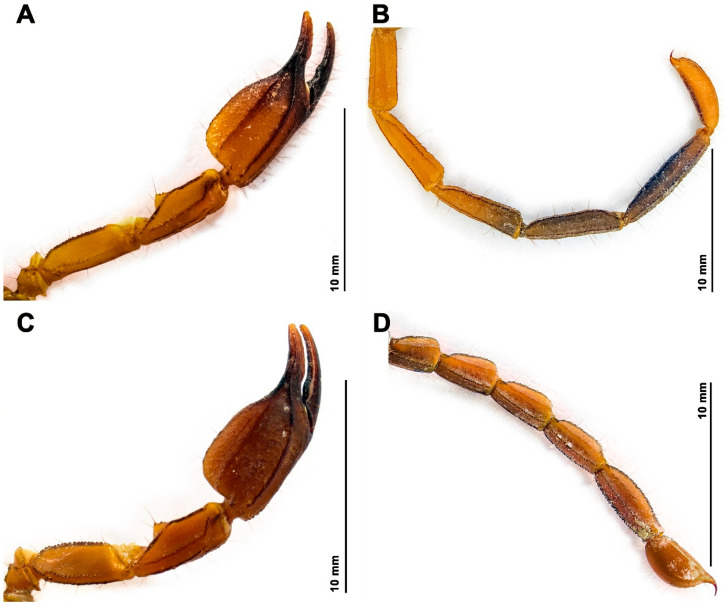
*Hemiscorpius aratta* sp. n., dextral pedipalp, dorsal aspect (**A**,**C**) and metasomal segments I–V and telson, lateral aspect (**B**,**D**): (**A**,**B**) Holotype male (RIZ HD-30); (**C**,**D**) Paratype female (RIZ HD-28).

**Description.** Based on the holotype ♂ (RIZ [HD-30]) (Figure 6A, Figure 7A,B, Figure 8 and Figure 9A,B, Table 5) with differences in the female noted where applicable.

*Total length*: Medium-sized, 68.9 mm.

*Color*: Base color brown (Figure 7); chelicerae yellow, teeth of fingers dark brown; carapace brown with dark spots (Figure 8A), median and lateral ocular tubercles black; pedipalp trochanter and femur yellow, patella brown, carinae darker, chela dark brown with reddish-brown fingers (Figure 9A); legs yellow (Figure 6A and Figure 7), femur darker, ungues darker; tergites dark brown, III–VI becoming paler posteriorly, VII paler (Figure 6A and Figure 7A); sternites dark yellow, becoming paler posteriorly (Figure 7B and Figure 8B); metasomal segments brown, segments III–V darker than I and II, carinae darker (Figure 7A,B and Figure 9B); telson vesicle, dorsal and ventral surfaces reddish-brown, lateral surfaces yellow, aculeus reddish-brown (Figure 9B). 

*Chelicerae*: Fixed finger median and basal teeth bifurcate; movable finger with one subdistal tooth and one basal tooth; teeth without secondary serrations.

*Carapace*: Carapace subrectangular (Figure 8A); frontal notch semicircular; lateral margins each with four or five small granules ventral to lateral ocelli; anteromedian longitudinal sulcus narrow, bifurcate anteriorly, continuous from anterior margin to median ocular tubercle; posteromedian sulcus forming deep depression; posterolateral sulcus shallow (Figure 8A); surfaces finely granular with three smooth areas, longer than wide, near lateral ocelli; median ocular tubercle situated in anterior half of carapace (Figure 8A), superciliary carinae smooth; three pairs of lateral ocelli, posterior ocellus smaller than others.

*Pedipalps*: Femur elongate, 2.9 times longer than wide (Table 5); pentacarinate (Figure 9A), with four distinct carinae; prodorsal, proventral, retrodorsal, and retroventral carinae each comprising large granules; ventromedian carina reduced to few granules proximally; dorsal surface finely granular; prolateral surface with few prominent spiniform granules; retrolateral surface shagreened; ventral surface finely and sparsely granular. Patella elongate, 2.8 times longer than wide (Table 5); seven carinae (Figure 9A), six distinct; prodorsal carina comprising large isolated granules; retrodorsal carina obsolete, granular; proventral, retroventral, and retromedian carinae granular; dorsal and ventral surfaces smooth; prolateral process pronounced, comprising prodorsal and proventral tubercles, prodorsal tubercle with single spiniform granule. Chela stout (Figure 9A), manus 1.65 times longer than wide (Table 5); five distinct carinae; dorsomedian and retromedian carinae granular; prodorsal carina coarsely granular; retrodorsal carina almost smooth; proventral carina comprising several spiniform granules; manus dorsal and ventral surfaces smooth; movable finger longer than manus (Table 5); fixed finger with proximal notch; movable finger with median lobe; movable finger with pro- and retrolateral rows of denticles; fixed finger with retrolateral denticle row complete, prolateral denticle row comprising separate granules merging proximally into row near fourth median denticle; fingertips each with pronounced terminal hook. Trichobothrial pattern Type C.

*Legs*: Ventral surfaces smooth; dorsal surfaces of trochanter and femur of legs II–IV sparsely granular; telotarsal ungues of equal length.

*Sternum*: Sternum pentagonal, longer than wide (Figure 8B); median sulcus deep, more pronounced in posterior half; posterior depression absent.

*Genital operculum*: Genital operculum comprising two subtriangular sclerites (Figure 8B); genital papillae short, not protruding from beneath operculum.

*Pectines*: Pectines reaching to or extending past distal margin of leg IV trochanter; posterior margins with 15/15 teeth (Figure 8B).

*Mesosoma*: Tergites granular, each with shallow median to submedian depressions; median carina absent from tergites I and II, obsolete on III and VII; tergite VII wider than long, lateral and sublateral carinae granular, restricted to posterior three-quarters. Sternites III–VI smooth, each with pair of deep median sulci; sternite VII wider than long, smooth, with pair of lateral carinae, median carina obsolete; spiracles crescent-shaped.

*Metasoma*: Segments elongate and slender (Figure 7 and Figure 9B); surfaces smooth. Segments I–IV each with seven carinae; dorsosubmedian and median lateral carinae granular; ventrolateral carinae smooth on segments I and II, granular on III and IV; ventromedian carinae granular. Segment V with five carinae; dorsosubmedian carinae granular; median lateral carinae obsolete, each with sparse row of granules in anterior two-thirds; ventrolateral and ventromedian carinae each comprising spiniform granules.

*Telson*: Vesicle elongate (Figure 9B), length/width = 4.3 (Table 5); pair of blunt tubercles posteriorly at base of aculeus; posterior tubercles each with small spiniform granules; dorsal surface granular; lateral surface sparsely granular; ventral surface granular with few macrosetae anteriorly, more numerous near base of aculeus; aculeus short, stout, strongly curved, narrower medially (Figure 9B).

**Intraspecific variation.** Males and females differ as follows. Total length 49.7–68.9 mm (♂), 38.8–48.4 mm (♀) (Table 5). Males are darker in color than females (Figure 7). The genital operculum comprises one (♀) or two (♂) sclerites with genital papillae present (♂) or absent (♀) beneath. The pectines bear 14–15 (♂) or 8–10 (♀) teeth (Table 5). The metasomal segments are longer in the male (Figure 7A and Figure 9B) than the female (Figure 7C and Figure 9D). The telson vesicle is elongate with a pair of blunt tubercles posteriorly at the base of the aculeus in the adult male (Figure 9B) but oval and without tubercles in the adult female (Figure 9D) and the immature stages.

**Figure 10:**
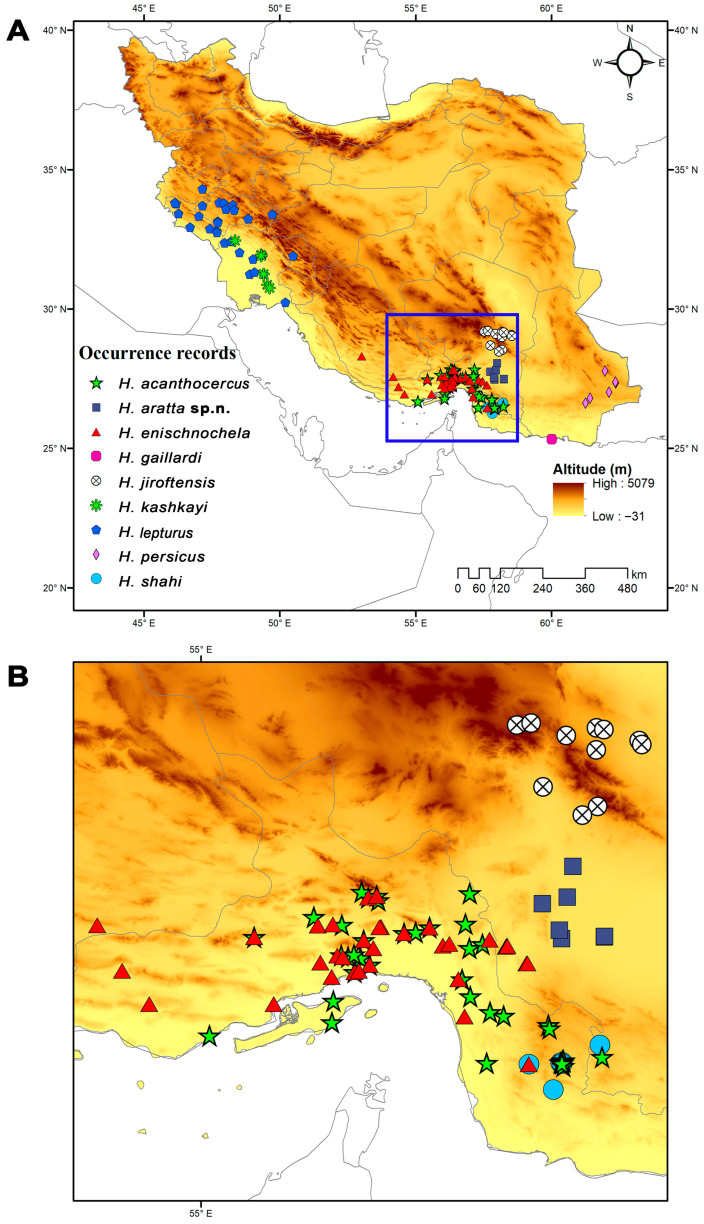
(**A**) Known locality records of nine species of *Hemiscorpius* Peters, 1861 recorded from Iran, indicating area enlarged in (**B**). (**B**) Enlarged view of southern Iran, illustrating overlapping ranges of *Hemiscorpius* species. Topographical elevation map obtained from the Shuttle Radar Topography Mission (SRTM) elevation model.

**Distribution.** Known only from lowlands (400–600 m) in the south of Kerman Province, southern Iran (Figure 10). The closest relative, *H. jiroftensis*, inhabits highlands.

***Hemiscorpius enischnochela* Monod & Lourenço, 2005**

*Hemiscorpius enischnochela* Monod & Lourenço, 2005: 886–896, Figures 1E, F, 8–12, 26A, B, 27C, D, 36; Navidpour et al., 2013: 17, 18, Figure 12; Salari & Sampour, 2017: 103, Figures 3–6; Shahi & Barahoei, 2023: 1593–1595, Figures 4–6, 7B; Adeli-Sardou et al., 2024: 647, Figures 2, 3.

**Material examined. IRAN:** *Fars Prov.*: Lar, 27°45′10″ N, 53°46′18″ E, 11/II/2021, H. Dehghan & E. Amiri, 1♂, 1♀ (JMU [HD-15], 1♂ [HD-19]). *Hormozgan Prov*.: Bandar Abbas district, 27°14′57.4″ N 56°21′16.3″ E, 2013, M. Heydaripour, 2♂, 2♀ (RIZ [Hem-63]), 2021, S. Khadir, 1♂, 1♀ (RIZ [Hem-66]); Fin, Tal-e Gerdu village, 27°48′14.7″ N 56°24′39.9″ E, 2013, M. Dehghan, 1♂, 1♀ (RIZ [Hem-153]); Genow village, 27°26′47″ N 56°18′20.2″ E, 25/IV/2014, M. Shahi, 6♂, 4♀ (RIZ [Hem-08]), 27/X/2022, H. Barahoei & M. Shahi, 2♂ (RIZ [Hem-156]); Keshar-e Oliya, 27°16′01.7″ N 55°57′31.5″ E, 2013, K. Karjou, 2♂, 1♀ (RIZ [Hem-55]); Khorgo village, 27°33′09.7″ N 56°26′36.5″ E, 2013, Behrouzi, 1♂, 1♀ (RIZ [Hem-124]); Konaru, 27°18′39.9″ N 56°08′16.4″ E, 2013, A. Mahyari, 2♂, 1♀ (RIZ [Hem-48]); Rezvan, 27°34′21.6″ N 56°03′29.6″ E, 2013, A. Dastpak, 2♂ (RIZ [Hem-58]); Shamil, 27°32′56.2″ N 56°50′10″ E, 2013, Sh. Speed, 5♂ 4♀ (RIZ [Hem-59]); Siyahu, 27°47′21.3″ N 56°20′27.9″ E, 2013, S. Kamali, 3♂, 2♀ (RIZ [Hem-60]); Takht, 27°30′13.9″ N 56°37′50.6″ E, 2013, M.A. Khodajouyan, 1♂, 1♀ (RIZ [Hem-46]); Khamir Co.: 2♂, 1♀ (RIZ [Hem-47]); Khamir River, 2013, A. Gorikhteh, 1♂, 1♀ (RIZ [Hem-51]); Lavar, 27°33′48.2″ N 55°56′19.5″ E, 2014, K. Khedmati, 2♂, 2♀ (RIZ [Hem-115]); Ruydar, 27°28′18.8″ N 55°25′38.1″ E, 2011, M. Mirkarimi, 2♂, 2♀ (RIZ [Hem-03]).

**Distribution.** Endemic to Fars, Hormozgan and Kerman provinces of southern Iran [6].

***Hemiscorpius jiroftensis* Amiri Ghanat Saman et al., 2025**

*Hemiscorpius jiroftensis* Amiri Ghanat Saman et al., 2025: 4–11, Figures 1–7.

*Hemiscorpius lepturus* Peters, 1861a: Navidpour et al., 2011: 21, Figures 7, 16, 65–68.

**Material examined. IRAN:** *Kerman Prov.*: Jiroft, Hishin village, 28°36′40″ N 57°31′31″ E, 10/IX/2024, H. Dehghan & E. Amiri, holotype ♂ (RIZ [HD-44A]), 1♂, 1♀ paratypes (RIZ [HD-44B, C]); Bam, Dehbakri, 29°01′14″ N 57°58′38″ E, 27/VIII/2024, H. Dehghan & E. Amiri, 6♂, 2♀ paratypes (JMU [HD-23]).

**Distribution.** Endemic to the highlands of Kerman Province in southern Iran [7].

***Hemiscorpius lepturus* Peters, 1861**

*Hemiscorpius lepturus* Peters, 1861: 426, 8 figs.; Karsch, 1879: 15, 21; Birula, 1905a: 146; Birula, 1917: 215; Birula, 1918: 42, Figure 7; Weidner, 1959: 100; Pringle, 1960: 84, Figure 9; Khalaf, 1962: 2; Khalaf, 1963: 68; Habibi, 1971: 44; Vachon, 1966: 214; Pérez Minocci, 1974: 36; Vachon, 1977: 213; Vachon, 1979: 59; Farzanpay, 1987: 141, 168; Farzanpay, 1988: 42; Simard & Watt, 1990: 441; Sissom, 1990: 75; El-Hennawy, 1992: 135; Kovařík, 1997: 48; Kovařík, 1998: 136; Fet, 2000: 429; Prendini, 2000: 44; Capes & Fet, 2001: 303; Monod & Lourenço, 2005: 902, Figures 1a, b, 16–21, 27e, f, 36; Akbari, 2007: 76, fig. p. 68; Navidpour et al., 2008a, Figures 20, 21, 43, 107–110: 26; Navidpour et al., 2008b: 20, Figures 2, 5, 7, 24, 78–81; Navidpour et al., 2008c: 15, Figures 4, 12, 67–70; Navidpour et al., 2008d: 14, Figures 3, 6, 7, 9, 14, 56–59; Kovařík, 2009: 19; Pirali-Kheirabadi et al., 2009: 12, Figures 3, 11, 49–52; Navidpour et al., 2010: 17; Karataş et al., 2012: 118; Al-Asmari et al., 2013: 5; Pirali-Kheirabadi et al., 2013: 49; Mozaffari et al., 2013: 5414; Taherian et al., 2014: 48; Nazari & Rastegar, 2016: 7; Sharifinia et al., 2017: 245; Nejati et al., 2018: 7140; Mansouri et al., 2020: 765.

*Hemiscorpion lepturus*: Peters, 1861b: 511; Ausserer, 1880: 466; Kraepelin, 1899: 142; Werner, 1934: 276; Moritz & Fischer, 1980: 317; Kovařík, 2002: 14.

*Hemiscorpio lepturus*: Simon, 1880b: 29.

**Material examined. IRAN:** *Khuzestan Prov.*: Ahvaz, 31°52′86″ N 49°87′98″ E, 10/III/2024, H. Dehghan, 8♂, 6♀ (RIZ [HD-42]).

**Distribution.** Widespread in Iraq and western Iran (Bushehr, Ilam, Kermanshah, Khuzestan, and Lorestan provinces) [6,7,50].

***Hemiscorpius persicus* (Birula, 1903)**

*Hemiscorpion persicum* Birula, 1903: 77–80.

*Hemiscorpius persicus*: Monod & Lourenço, 2005: 913–921, Figures 22–25; Barahoei et al., 2020b: 418, Figures 32, 33; Moradi et al., 2020: 94–95, Figures 8–9.

*Habibiella persica*?: Farzanpay, 1990: 9.

*Habibiella persicus*: Farzanpay, 1987: 169, 170.

**Material examined. IRAN:** *Sistan & Baluchistan Prov.*: Sarbaz, Baftan village, 26°47′18″ N 61°24′34″ E, 29/X/2019, N. Behi, 1♂ (ZMBK [MM-25]); Saravan, 27°22′23.2″ N 62°21′14.3″ E, 4/V/2024, F. Vahidinia, 1♀ (RIZ [FV-25]); Dezek village, 27°20′53.4″ N 62°21′37.5″ E, 25/VII/2022, A. Hosseinbor, 1♀ (ZMBK [MM-19]); Poujgeli village, 27°46′13.9″ N 61°58′00.4″ E, 25/IX/2022, A. Hosseinbor, 1♂ (ZMBK [MM-20]); Hidouj village, 27°00′28.2″ N 62°06′53″ E, 2/X/2022, A. Hosseinbor, 1♀ (ZMBK [MM-21]).

**Distribution.** Endemic to Sistan & Baluchistan Province in southeastern Iran [6,48].

## 4. Discussion

### 4.1. Morphological Variation

The Iranian species of *Hemiscorpius* may be divided into two groups based on overall body size, pedipalp length, and the number of trichobothria on the ventral surface of the pedipalp patella. The first group comprises six species which are smaller in body size, with shorter pedipalps, and three trichobothria on the ventral surface of the pedipalp patella [7]: *H. acanthocercus*; *H. aratta* sp. n.; *H. jiroftensis*; *H. kashkayi*; *H. lepturus*; *H. persicus*.

The three species comprising the second group are larger in body size, with elongate, slender pedipalps, and more than ten trichobothria on the ventral surface of the pedipalp patella: *H. enischnochela*; *H. gaillardi*; *H. shahii*. *Hemiscorpius gaillardi*, originally assigned to the monotypic genus *Habibiella* Vachon, 1974, was described from a single female without precise locality data. It is probably restricted to the southern Makkoran Mountains but has not been recollected since the original description. Reports from Fars [51] and Hormozgan [52] provinces are probably misidentifications. The largest species in the genus, *H. shahii*, with the highest count (more than 14) of trichobothria on the ventral surface of the pedipalp patella, is restricted to the highlands of eastern Hormozgan Province [6]. *Hemiscorpius enischnochela* occurs in central to western Hormozgan Province and southern Kerman Province, its distribution overlapping that of *H. acanthocercus*. Although sympatric in some areas, *H. acanthocercus* and *H. enischnochela* are clearly distinct based on morphometric and molecular analyses (Figure 2 and Figure 3, Table 1).

The morphological differences between the two groups of Iranian *Hemiscorpius* suggest that they could be distinct genera, in which case, *Habibiella* would need to be revalidated from synonymy with *Hemiscorpius* [15]. However, this proposal is unsupported by the position of *H. enischnochela* recovered in the molecular phylogenetic analyses presented here (Figure 3). A resolution of this question requires more data, including the acquisition of DNA sequences from *H. gaillardi* and *H. shahii*.

Previous studies reported that *H. persicus* is not sexually dimorphic [15,52,53]. However, the present study revealed long, slender metasomal segments and an elongate telson with a pair of blunt tubercles posteriorly at the base of the aculeus, in adult males of *H. persicus*. The alleged absence of dimorphism in earlier reports was likely caused by mistaking subadult males for adults. Based on the collection localities and images published by Moradi et al. [54], male specimens misidentified as *H. lepturus* are evidently conspecific with *H. persicus*. Reports of *H. persicus* from Hormozgan Province [55] are likely also misidentifications, mistakenly attributed to the absence of sexual dimorphism.

The validity of *H. kashkayi*, another species allegedly not sexually dimorphic [16], is unclear. Since its initial description, *H. kashkayi* has not been reported or recollected, and it might be synonymous with *H. lepturus*.

### 4.2. Morphometric Analyses

Sexual dimorphism is evident as differences in body size and the shape of structures [26]. Sexual size dimorphism is common in scorpions [26,29]. The males of many scorpion species are smaller, with a more elongated metasoma and a larger telson, than conspecific females [26,28,56,57].

In the present study, sexual dimorphism was observed in overall body size (Type II ANOVA) as well as in the shape of structures (Type II MANOVA). The metasoma and telson were longer in males but wider in females, even among adults of the same size (Table 1).

Type II ANOVA revealed significant differences in the interaction between sex and species suggesting that sexual size dimorphism evolved in different directions. Overall body size was greater in females of all species except *H. lepturus* in which it was greater in males (Figure 1). Type II MANOVA indicated significant differences between the sexes and significant differences in the interaction effects, suggesting that shape may be under strong selection.

Sexual dimorphism was evident in all species according to the box plot analyses. *Hemiscorpius acanthocercus* was the smallest species (Figure 1), based on the univariate analyses, with or without sexual dimorphism (Table 2, Table 3 and Table 4), and was separated from other species along LD1 by the shape of the pedipalp chela manus (Figure 2) in the multivariate analysis (LDA). The multivariate analysis also separated *H. aratta* sp. n. from the closely related *H. jiroftensis* along LD2, based on the shorter pedipalp chela movable finger relative to the chela manus, a difference also evident in the univariate analysis (Table S2). Significant differences were also evident between *H. lepturus* and the other species along LD2 in the multivariate analysis (Figure 2) and in the boxplots of overall body size, reinforcing that, unlike other species, overall body size was greater in males than females.

### 4.3. Molecular Analyses

Phylogenetic analysis and genetic distances supported the monophyly of all species and confirmed the validity of the new species (Figure 3; Table 3). Despite forming a monophyletic group in the phylogenetic analysis (Figure 3), the genetic distance between samples of *H. enischnochela* from Fars Province and Hormozgan Province was greater (0.051) than usually observed for intraspecific variation [7,19]. The genetic distance between the samples from Khamir and Lar and the other samples of *H. enischnochela* was 0.070, supporting the morphometric analysis, in which the specimens from Khamir and Lar were also well separated from other conspecifics (Figure 2).

*Hemiscorpius lepturus* formed a distinct clade, sister to all other species, in the phylogeny (Figure 3). The highest genetic distances were observed between *H. lepturus* and the other species (Table 3). Additionally, the intraspecific distances in *H. lepturus* were relatively high (0.068) (Table 3), suggesting the possibility of cryptic diversity in southwestern Iran. Samples from Iraq and Lorestan Province grouped sister to the population from Khuzestan Province. Within Khuzestan Province, samples from the highlands (Dezful, Izeh, and Ramhormoz) were separated from the lowland sample (Ahvaz), each forming sister clades (Figure 3). *Hemiscorpius lepturus* is widespread across the region, extending from Iraq to the lowlands of Lorestan Province in Iran. Populations from the highlands of Khuzestan and Lorestan provinces may prove to be conspecific with *H. kashkayi*, if indeed the latter species is valid. This question will remain unresolved, however, until topotypes of *H. kashkayi* are collected and sequenced. On the other hand, populations from the lowlands of Bushehr and Khuzestan provinces may represent an undescribed species. The limits of *H. lepturus* and the validity of *H. kashkayi* await further investigation with a more comprehensive taxon sample.

GenBank sequences from Izeh (KU341987) and Ramhormoz (KU341988), respectively identified as *H. lepturus* and *H. persicus* (Table 2), are genetically identical and evidently conspecific with *H. lepturus*, based on their collection localities (Khuzestan Province). The present study provides the first DNA sequences of *H. persicus* based on accurately identified samples.

Genetic distances among the geographically adjacent species, *H. aratta* sp. n., *H. enischnochela*, and *H. jiroftensis*, were similar (0.078) (Table 3), supporting their recognition. The lowest genetic distance was obtained between the new species and *H. persicus* (0.067, Table 3). However, the two species are well separated morphologically and geographically (Figure 10).

Additional sampling will be necessary to resolve the phylogenetic positions and taxonomic status of other Iranian species of the genus, e.g., *H. gaillardi* and *H. shahii*.

### 4.4. Iranian Biogeography

Geographical barriers play a crucial role in scorpion speciation [3,7,27,28]. The interactions between landmasses, which led to the formation of the Jebal Barez, Makkoran, and Zagros mountains, led to the separation of *Hemiscorpius* populations during different geological time periods, ultimately resulting in speciation.

Based on the phylogenetic tree, it may be inferred that the Zagros Mountains separated the western populations (*H. kashkayi* and *H. lepturus*) from the populations in central Iran. The population inhabiting Hormozgan Province (*H. enischnochela*) was isolated from the lowland population in the southern half of Kerman Province (*H. aratta* sp. n.) by the mountains of that region. Similarly, the population inhabiting the mountainous Jebal Barez range (*H. jiroftensis*) is distinct from the lowland population, described herein as *H. aratta* sp. n. The Makkoran Mountains separated the population to the north of this range (*H. persicus*) from the population at the southeastern end of the Zagros Mountains (*H. acanthocercus*). Although no DNA sequences are currently available for several Iranian species of *Hemiscorpius*, it is likely that the populations inhabiting the northern (*H. persicus*) and southern (*H. gaillardi*) slopes of the Makkoran Mountains are also isolated from each other. Similarly, the population inhabiting the highlands where the Makkoran and Zagros ranges converge (*H. shahii*) appears to be separated from *H. enischnochela*. These preliminary hypotheses await testing in a rigorous biogeographical analysis with molecular divergence dating.

### 4.5. Ecological Niche Modeling

Consistent with their preference for warm, humid habitats, the Iranian species of *Hemiscorpius* are distributed primarily in the southern half of the country, particularly along the coasts of the Gulf of Oman and the Persian Gulf (Figure 10) [50]. Ecological niche modeling across Iran reveals that temperature extremes, particularly low winter temperatures and seasonal precipitation patterns, are the primary factors influencing the distributions of *Hemiscorpius* species. These climatic variables restrict species like *H. acanthocercus* to the warm coastal lowlands of Iran, suggesting a narrow climatic tolerance and high susceptibility to climate change. Rising temperatures could potentially relax these constraints, allowing for range expansion, or intensify competition with other scorpions. The distributions predicted for several species, including *H. aratta* sp. n., highlight the importance of coastal habitats, especially in Hormozgan Province, where climatic factors such as annual temperature range (BIO7) create favorable conditions for species not yet recorded. Despite limited occurrence data, niche models suggest these areas should be targeted for future field surveys to refine species distributions and improve risk mapping, as climate change is likely to shift habitats.

Although high AUC values indicate strong model discrimination, their interpretation in presence-only models with relatively small sample sizes should be treated with caution. Model outputs are best viewed as hypotheses of potential habitat suitability rather than definitive range boundaries.

Microhabitat conditions, e.g., rocky substrates, temperature seasonality, and precipitation patterns, are crucial for the persistence of species with specialized habitat requirements, such as *H. enischnochela*, *H. jiroftensis*, and *H. kashkayi*. These species are vulnerable to climate change and human land-use modifications. For example, *H. kashkayi*, appears to be restricted to cooler environments in the Zagros Mountains, where its tolerance to high summer temperatures (BIO5) is a critical factor shaping its distribution. Similarly, *H. enischnochela* relies on stable coastal conditions with specific moisture availability, whereas *H. jiroftensis* is confined to semi-arid climates, emphasizing the role of seasonal precipitation and temperature variation in habitat suitability. The narrow ecological niches and limited habitat suitable for these species underline their vulnerability to environmental change. For example, *H. persicus* is dependent on seasonal precipitation (BIO16) and stable temperatures (BIO3), making it particularly sensitive to shifts in climate patterns. The model predicts that only a small fraction of Iran provides suitable habitat for *H. persicus*, underscoring the importance of focused conservation efforts. Similarly, *H. shahii* is restricted to warm coastal regions, where maritime climates and temperature seasonality provide the necessary conditions for survival. This species is highly sensitive to aridity, and inland environments lack appropriate microhabitats. The range of *H. shahii* appears to be more limited than initially predicted, reinforcing the need for targeted conservation in coastal areas under threat from urban development and climate change.

In general, the findings presented for *Hemiscorpius* are consistent with previous studies of scorpion distribution in Iran, which identified the southern and southeastern regions as biodiversity hotspots [6,7,19,50]. Projected climate changes, including rising minimum temperatures and altered precipitation, may further restrict or shift the ranges of *Hemiscorpius* species, emphasizing the need for continued monitoring, high-resolution ecological surveys, and risk mapping to inform conservation strategies and anticipate potential human–scorpion interactions [5,58,59]. Understanding the ecological constraints of *Hemiscorpius* species is also important for public health planning, especially in areas prone to scorpionism. To mitigate the risks associated with scorpion envenomation, targeted interventions, such as antivenom distribution and public awareness campaigns, should focus on habitats identified as suitable for medically important taxa.

## 5. Conclusions

An understanding of the taxonomic limits, diagnostic characters, and geographical distributions of the species of *Hemiscorpius* is essential because of their medical importance. For this reason, a survey of the scorpions in this genus was conducted in southern Iran, which resulted in the identification of a new species from the southern part of Kerman Province, described herein as *Hemiscorpius aratta* sp. n. Following this discovery, the number of species of *Hemiscorpius* recorded in Iran has increased to nine, most of which occur in the southern half of the country.

## Figures and Tables

**Table 1:** *p* values from pairwise comparisons among males (m) and females (f) of six Iranian species of *Hemiscorpius* Peters, 1861 using Welch’s two-sample *t*-test: * (0.05–0.01); ** (0.01–0.001); *** (<0.001); ns (nonsignificant). CL: carapace length; CW: carapace width; FL: pedipalp femur length; FW: pedipalp femur width; PL: pedipalp patella length; PW: pedipalp patella width; ML: pedipalp chela manus length; MW: pedipalp chela manus width; MFL: pedipalp chela movable finger length; MSL: mesosoma length; MSW: mesosoma tergite IV width; MT1L, MT2L, MT3L, MT4L, MT5L: length of metasomal segments I–V; MT1W, MT2W, MT3W, MT4W, MT5W: width of metasomal segments I–V; TL: telson length; BTL: body total length.

Character	Species					
	*H. acanthocercus*	*H. aratta* sp. n.	*H. enischnochela*	*H. jiroftensis*	*H. lepturus*	*H. persicus*
CL	f *	ns	ns	ns	ns	ns
CW	ns	ns	ns	ns	ns	ns
FL	ns	ns	ns	ns	ns	ns
FW	f *	ns	ns	ns	ns	ns
PL	ns	ns	ns	ns	ns	ns
PW	ns	ns	ns	ns	ns	ns
ML	ns	ns	ns	ns	ns	ns
MW	ns	ns	ns	ns	ns	ns
MFL	ns	ns	ns	ns	ns	ns
MSL	ns	ns	ns	ns	ns	ns
MSW	f **	f *	ns	ns	ns	ns
MT1L	m ***	m ***	m ***	ns	m ***	ns
MT1W	f ***	f *	ns	ns	ns	ns
MT2L	m ***	m ***	m ***	m *	m ***	m *
MT2W	f ***	f *	ns	ns	f *	ns
MT3L	m ***	m ***	m ***	m **	m ***	m *
MT3W	f ***	f *	ns	ns	f **	ns
MT4L	m ***	m ***	m ***	m *	m ***	m *
MT4W	f ***	f **	ns	ns	f **	ns
MT5L	m ***	m ***	m ***	ns	m ***	m *
MT5W	f **	f *	ns	ns	f *	ns
TL	m *	m *	ns	ns	m *	ns
BTL	m ***	m ***	m ***	ns	m *	ns
CL/W	ns	ns	ns	ns	ns	ns
FL/W	ns	ns	ns	ns	ns	m *
PL/W	ns	m *	ns	ns	ns	ns
ML/W	ns	ns	ns	ns	m *	ns
MFL/ML	ns	ns	ns	ns	ns	ns
MSL/MSW	m **	ns	m *	ns	ns	m *
MT1L/W	m ***	ns	m ***	m ***	m ***	ns
MT2L/W	m ***	m ***	m ***	m ***	m ***	m **
MT3L/W	m ***	m ***	m ***	m **	m ***	m *
MT4L/W	m ***	m ***	m ***	m ***	m ***	m ***
MT5L/W	m ***	m ***	m ***	m **	m ***	m *

**Table 2:** Specimen repositories, sample codes, locality data and GenBank accession codes for samples of *Hemiscorpius* Peters, 1861 and the outgroup, *Scorpio palmatus* (Ehrenberg, 1828), used for DNA sequencing. Material is deposited in the following collections: Ambrose Monell Cryocollection (AMCC) at the American Museum of Natural History, New York; the Medical Entomology Collection of Jiroft University of Medical Science, Iran (JMU); the Research Institute of Zabol, Iran (RIZ); and the Zoological Museum of Shahid Bahonar University of Kerman, Iran (ZMBK).

Species	Collection	Locality	GenBank	Reference
*H. acanthocercus*	RIZ [Hem-098A]	IRAN: *Hormozgan Prov*.: Bandar Abbas	OR803737	[19]
	RIZ [Hem-098B]		OR803738	
	RIZ [Hem-151]		OR803739	
*H. aratta* sp. n.	RIZ [HD-28]	IRAN: *Kerman Prov.*: Kahnouj	PX640594	This study
	JMU [HD-33]		PX640595	
	JMU [HD-34]		PX640596	
	JMU [HD-36]		PX640597	
*H. enischnochela*	RIZ [Hem-098A]	IRAN: *Hormozgan Prov.*: Parsian	OR828045	[19]
	RIZ [Hem-098A]		OR828046	
	RIZ [Hem-148]	IRAN: *Hormozgan Prov.*: Bandar Abbas	OR828047	
	RIZ [Hem-098]		OR828048	
	RIZ [Hem-087]	IRAN: *Hormozgan Prov.*: Khamir	OR828049	
	JMU [HD-15]	IRAN: *Fars Prov.*: Lar	PX608632	This study
	JMU [HD-19]		PX608633	
*H. jiroftensis*	JMU [HD-23]	IRAN: *Kerman Prov.*: Bam	PX057766	[7]
	JMU [HD-45A]		PX057767	
	JMU [HD-45B]		PX057768	
	RIZ [HD-44A]	IRAN: *Kerman Prov.*: Jiroft	PX057769	
	RIZ [HD-44B]		PX057770	
*H. lepturus*	RIZ [Hem-162]	IRAN: *Khuzestan Prov.*: Dezful	OR800364	[19]
	-	IRAQ	MT230874	
	-	IRAN: *Khuzestan Prov.*: Izeh	KU341987	
	-	IRAN: *Khuzestan Prov.*: Ramhormoz	KU341988	
	-	IRAN: *Khuzestan Prov.*	OP433761	Unpublished
	-	IRAN: *Lorestan Prov.*	OP433762	
	RIZ [HD-42A]	IRAN: *Khuzestan Prov.*: Ahvaz	PX608629	This study
	RIZ [HD-42B]		PX608630	
	RIZ [HD-42C]		PX608631	
	RIZ [HD-42H]		PX608628	
*H. persicus*	ZMBK [MM-19]	IRAN: *Sistan & Baluchistan Prov.*: Saravan	PX608625	
	ZMBK [MM-20]		PX608626	
	ZMBK [MM-21]		PX608627	
*S. palmatus*	AMCC 101706	EGYPT	AY156585	[49]

**Table 3:** Average Kimura 2-parameter (K2P) genetic distances within (bold) and between six Iranian species of *Hemiscorpius* Peters, 1861 based on Cytochrome *c* Oxidase Subunit I sequences.

Species	1	2	3	4	5	6
*H. acanthocercus* (1)	**0.027**					
*H. aratta* sp. n. (2)	0.078	**0.000**				
*H. enischnochela* (3)	0.116	0.078	**0.051**			
*H. jiroftensis* (4)	0.120	0.078	0.093	**0.013**		
*H. lepturus* (5)	0.136	0.103	0.133	0.122	**0.068**	
*H. persicus* (6)	0.100	0.067	0.096	0.090	0.128	**0.003**

**Table 4:** Percent contribution of bioclimate modeling for eight Iranian species of *Hemiscorpius* Peters, 1861. Models indicated by—not used in prediction maps. Variables not influencing species distributions indicated by zero (0).

Variables		*H. acanthocercus*	*H. aratta* sp. n.	*H. enischnochela*	*H. jiroftensis*	*H. kashkayi*	*H. lepturus*	*H. persicus*	*H. shahii*
BIO2	Mean diurnal range, i.e., mean of monthly (maximum temperature – minimum temperature)	28.5	-	-	-	0	3.1	-	-
BIO3	Isothermality (BIO2/BIO7 × 100)	0.9	0	2.1	0	0.2	-	39.6	-
BIO4	Temperature seasonality	24.7	-	-	38.1	-	0.1	-	81.3
BIO5	Max temperature of warmest month	-	0	5.7	-	87.3	-	0	-
BIO6	Min temperature of coldest month	32.5	-	65	-	-	-	-	-
BIO7	Temperature annual range (BIO5, BIO6)	-	74.5	-	3.6	0.8	-	-	-
BIO9	Mean temperature of driest quarter	0.1	-	-	-	-	-	7.3	3.4
BIO10	Mean temperature of warmest quarter	0.8	-	2.6	-	-	-	-	-
BIO11	Mean temperature of coldest quarter	-	-	-	-	-	-	22.7	-
BIO12	Annual precipitation	0.8	-	0.4	32.5	-	1.2	-	-
BIO13	Precipitation of wettest month	-	-	-	-	9.2	0.3	-	-
BIO14	Precipitation of driest month	-	-	-	3.6	-	23.6	-	6
BIO15	Precipitation seasonality	-	21.1	-	-	-	0.4	-	8.8
BIO16	Precipitation of wettest quarter	-	4	-	-	-	-	28.6	-
BIO17	Precipitation of driest quarter	-	-	-	-	-	4.2	-	-
BIO18	Precipitation of warmest quarter	-	-	-	18.5	-	0	-	-
BIO19	Precipitation of coldest quarter	11.1	-	23.7	-	-	66.9	-	-
Slope		0.4	0.4	0.5	3.8	2.4	0.1	1.9	0.4

**Table 5:** Morphometric data for *Hemiscorpius aratta* sp. n. type specimens from Kerman Province, southern Iran. Measurements in mm. Material deposited in the Medical Entomology Collection of Jiroft University of Medical Science, Iran (JMU) and the Research Institute of Zabol, Iran (RIZ). Abbreviations: HT = holotype; PT = paratype.

		Specimen	JMU	JMU	RIZ	RIZ	RIZ	RIZ	JMU	JMU	JMU	RIZ	JMU	JMU	JMU	JMU	JMU
			14	14	28	28	31	30	35	37	14	28	33	34	36	36	40
Character			PT	PT	PT	PT	PT	HT	PT	PT	PT	PT	PT	PT	PT	PT	PT
			♂	♂	♂	♂	♂	♂	♂	♂	♀	♀	♀	♀	♀	♀	♀
Total body		L	58.6	57.9	65.5	59.2	49.7	68.9	60.8	57.23	39.6	48.3	48.4	44.7	44.23	38.81	44.86
Carapace		L	5.93	5.58	6.17	5.96	4.88	6.41	5.85	5.91	5.35	6.05	6.03	5.61	6.3	5.37	5.98
		ant. W	3.66	3.94	4.13	3.91	3.22	4.74	4.11	3.83	3.63	4.05	3.94	3.83	4.09	3.48	3.79
		post. W	4.94	4.98	5.18	4.67	4.02	5.66	5.08	4.76	4.85	5.13	4.89	5.11	5.23	4.43	4.89
Pedipalp	femur	L	5.07	5.14	5.47	5.11	4.1	6.21	5.53	5.25	4.58	5	5.14	4.77	4.94	4.45	4.91
		W	2.13	1.19	2.16	2.02	1.72	2.15	1.94	2.03	1.86	1.9	1.97	2.02	2.02	1.78	2.3
	patella	L	5.21	5.43	5.84	5.38	4.66	6.03	5.64	5.28	4.81	5.4	5.3	4.84	5.38	4.82	4.7
		W	2.25	1.98	2.36	2.25	1.96	2.12	2.41	2.08	2.14	2.28	2.31	2.36	2.31	2.07	1.99
	manus	L	5.15	5.14	5.58	5.37	4.42	6.16	5.52	5.32	4.8	5.9	5.31	5.38	5.62	4.69	5.4
		W	3.48	2.38	4	3.65	1.95	3.74	3.69	3.67	2.16	3.82	3.73	3.84	3.68	3.6	3.39
		D	2.27	3.51	2.26	2.16	3.18	2.65	2.29	2.18	3.75	2.36	2.08	2.66	2.48	1.94	1.97
	mov. finger	L	5.06	5.07	5.39	5.2	4.27	5.92	5.33	5.02	4.61	5.7	5.2	5.14	5.4	4.44	5.27
Mesosoma		L	13.41	12.8	16.3	15.2	13.5	16.6	14.7	13.98	12.3	16.1	17.5	15.6	12.35	11.66	17.34
Metasoma I		L	6.16	5.5	6.69	5.19	4.41	6.07	6.1	5.28	3.04	3.02	3.18	2.83	3.56	3.07	2.87
		W	1.8	1.8	1.84	1.68	1.55	1.84	1.73	1.48	1.82	1.96	1.97	1.95	2.04	1.95	1.68
		D	1.73	1.74	188	1.72	1.48	1.72	1.63	1.45	1.59	1.7	1.65	1.64	1.62	1.57	1.53
Metasoma II		L	6.65	6.31	7.34	5.96	5.22	7.2	6.56	5.9	3.27	3.39	3.66	3.49	3.81	3.16	3.08
		W	1.74	1.58	1.67	1.52	1.3	1.54	1.32	1.27	1.62	1.75	1.74	1.73	1.76	1.62	1.49
		D	1.69	1.76	1.72	1.58	1.47	1.61	1.57	1.54	1.56	1.67	1.65	1.67	1.6	1.65	1.67
Metasoma III		L	6.92	6.48	7.65	6.71	5.07	7.88	6.79	6.1	3.11	4.04	3.95	3.78	4.29	3.36	3.58
		W	1.61	1.46	1.49	1.45	1.39	1.54	1.29	1.2	1.76	1.5	1.6	1.62	1.63	1.51	1.43
		D	1.68	1.73	1.8	1.6	1.48	1.73	1.68	1.51	1.46	1.68	1.69	1.64	1.57	1.61	1.63
Metasoma IV		L	6.8	7.05	8.17	6.88	5.47	7.88	7.13	6.71	3.68	5.26	4.12	3.79	3.94	3.18	3.58
		W	1.33	1.25	1.19	1.13	1.09	1.41	1.15	1.14	1.38	1.63	1.38	1.47	1.48	1.34	1.36
		D	1.63	1.62	1.55	1.49	1.46	1.8	1.6	1.59	1.59	1.35	1.66	1.65	1.69	1.51	1.62
Metasoma V		L	7.62	8.01	8.72	7.67	6.32	9.53	7.9	7.79	4.3	5.34	5.07	4.86	5.21	4.52	4.22
		W	1.66	1.38	1.18	1.23	1.04	1.39	1.18	1.2	1.39	1.66	1.78	1.51	1.52	1.38	1.38
		D	1.69	1.68	1.69	1.59	1.54	1.83	1.73	1.61	1.58	1.38	1.99	1.72	1.59	1.6	1.58
Telson		L	5.11	6.14	4.44	5.61	4.82	7.31	5.82	5.56	4.57	5.14	4.93	4.7	4.77	4.49	4.21
		W	1.53	1.62	1.73	1.43	1.35	1.7	1.45	1.44	1.71	2.11	1.83	1.9	1.94	1.83	1.75
		D	1.8	1.75	1.04	1.7	1.56	1.92	1.82	1.66	1.81	2.04	1.97	1.93	1.85	1.84	1.67
Pectinal teeth		count	14	15	14	14	14	15	14	15	8	9	8	9	10	9	10
Mov./fixed fingers		rows	6/6	6/7	7/8	7/6	7/7	9/8	6/8	7/7	7/8	6/7	7/7	8/8	7/7	8/8	6/6

## Data Availability

All data generated or analyzed during this study are included in the published article and its Supplementary Files. The original data presented in the study are openly available from GenBank (National Center for Biotechnology Information) at https://www.ncbi.nlm.nih.gov accessed on 25 November 2025. Additional datasets are available from the corresponding author upon request.

## Supplementary Materials

The following supporting information can be downloaded at: https://www.mdpi.com/article/10.3390/insects17010018/s1: Table S1. Statistics (mean ± standard deviation) for morphometric characters without sexual dimorphism in six Iranian species of *Hemiscorpius* Peters, 1861 based on ANOVA and Tukey posthoc HSD tests (<, << and <<< indicate *p*-values less than 0.05, 0.01, and 0.001, respectively). Abbreviations: ac = *H. acanthocercus*; ar = *H. aratta* sp. n.; en = *H. enischnochela*; ji = *H. jiroftensis*; le = *H. lepturus;* pe = *H. persicus*; Table S2. Statistics (mean ± standard deviation) for morphometric characters with sexual dimorphism in males of six Iranian species of *Hemiscorpius* Peters, 1861 based on ANOVA and Tukey posthoc HSD tests (<, << and <<< indicate *p*-values less than 0.05, 0.01, and 0.001, respectively). Abbreviations: ac = *H. acanthocercus*; ar = *H. aratta* sp. n.; en = *H. enischnochela*; ji = *H. jiroftensis*; le = *H. lepturus*; pe = *H. persicus*; Table S3. Statistics (mean ± standard deviation) for morphometric characters with sexual dimorphism in females of six Iranian species of *Hemiscorpius* Peters, 1861 (based on ANOVA and Tukey posthoc HSD tests (<, << and <<< indicate *p*-values less than 0.05, 0.01, and 0.001, respectively). Abbreviations: ac = *H. acanthocercus*; ar = *H. aratta* sp. n.; en = *H. enischnochela*; ji = *H. jiroftensis*; le = *H. lepturus;* pe = *H. persicus*; Table S4. Areas (km^2^) of habitat suitable for eight Iranian species of *Hemiscorpius* Peters, 1861.
