# Supplementary material for: Molecules, Morphometrics and Modeling of the Medically Important Genus Hemiscorpius Peters, 1861 (Scorpiones: Hemiscorpiidae) in Iran Reveal New Species from Kerman
Source: Insects. 2025 Dec 23;17(1):18. doi: 10.3390/insects17010018 (PMC12842565; doi:10.3390/insects17010018)
Supplement: Supplementary file 1 [file insects-17-00018-s001.zip › insects-3997223-supplementary.pdf]

Supplementary data

Table S1. Statistics (mean ± standard deviation) for morphometric characters without sexual dimorphism in six Iranian species of *Hemiscorpius* Peters, 1861 based on ANOVA and Tukey posthoc HSD tests (<, << and <<< indicate *p*-values less than 0.05, 0.01, and 0.001, respectively). Abbreviations: ac = *H. acanthocercus*; ar = *H. aratta* sp. n.; en = *H. enischnochela*; ji = *H. jiroftensis*; le = *H. lepturus;* pe = *H. persicus*.

Character	*H. acanthocercus* (n = 23)	*H. aratta* sp. n. (n = 15)	*H. enischnochela* (n = 26)	*H. jiroftensis* (n = 8)	*H. lepturus* (n = 11)	*H. persicus* (n = 5)	ANOVA	HSD
CW	4.35 ± 0.70	3.90 ± 0.34	5.88 ± 1.10	4.77 ± 0.35	4.31 ± 0.20	3.92 ± 0.58	F = 19.37 $p < e^{-11}$	ac=le=pe<<<en, ji<<en, ar<<<en
FL	4.50 ± 0.67	5.04 ± 0.49	6.91 ± 1.1	7.16 ± 0.95	5.54 ± 0.22	5.18 ± 0.62	F = 29.73 $p < e^{-15}$	ac <<<en=ji, ac<<le, le=pe=ar<<<en, le=pe=ar<<<ji
PL	4.55 ± 0.66	5.25 ± 0.41	6.76 ± 1.05	6.89 ± 0.48	5.64 ± 0.25	5.22 ± 0.49	F = 28.73 $p < e^{-15}$	ac <<<en=ji, ac<<pe, le=pe=ar<<<en, le=pe<<ji, ar<<<ji
PW	1.88 ± 0.28	2.19 ± 0.15	2.63 ± 0.40	2.56 ± 0.24	2.32 ± 0.18	2.19 ± 0.39	F =16.68 $p < e^{-10}$	ac <<<en=ji, ac<<pe, ac<ar, pe<en, ar<<<en
ML	8.97 ± 1.41	5.22 ± 0.42	12.11 ± 3.03	6.78 ± 0.68	5.59 ± 0.27	5.29 ± 0.49	F = 39.30 $p < e^{-15}$	ac<<<en, le=ar<<<ac, pe<<ac, ji=le=pe=ar<<<en
MW	3.37 ± 0.58	3.38 ± 0.65	3.40 ± 0.68	4.90 ± 0.68	4.12 ± 0.25	3.60 ± 0.68	F = 10.04 $p < e^{-6}$	ac<<en, ac=ar<<<ji, ac<le, en<<ji, ar<en=le, pe<<ji
MFL	4.15 ± 0.77	5.31 ± 0.40	6.54 ± 1.06	7.30 ± 0.75	5.70 ± 0.30	5.31 ± 0.68	F = 31.19 $p < e^{-15}$	ac<<en=ji=le=ar, ac<pe, le=pe<en, ar<<<en, le=pe=ar<<<ji, pe<<<le
MSL	12.18 ± 2.03	14.61 ± 1.92	16.43 ± 2.70	19.69 ± 3.70	16.26 ± 2.95	16.21 ± 3.47	F =12.75 $p < e^{-8}$	ac<<en=ji=le, a<pe, en<ji, ar<<<ji
CL/W	1.19 ± 0.03	1.50 ± 0.07	1.24 ± 0.19	1.53 ± 0.07	1.48 ± 0.05	1.51 ± 0.08	F = 26.4 $p < e^{-15}$	ac<<<ji=le=pe=ar, en<<<ji=le=pe=ar
MFL/ML	0.47 ± 0.08	1.02 ± 0.05	0.58 ± 0.24	1.08 ± 0.07	1.02 ± 0.03	1.00 ± 0.07	F = 56.12 $p < e^{-15}$	ac<<<ji=le=pe=ar, en<<<ji=le=pe=ar

Table S2. Statistics (mean ± standard deviation) for morphometric characters with sexual dimorphism in males of six Iranian species of *Hemiscorpius* Peters, 1861 based on ANOVA and Tukey posthoc HSD tests (<, << and <<< indicate *p*-values less than 0.05, 0.01, and 0.001, respectively). Abbreviations: ac = *H. acanthocercus*; ar = *H. aratta* sp. n.; en = *H. enischnochela*; ji = *H. jiroftensis*; le = *H. lepturus*; pe = *H. persicus*.

Character	*H. acanthocercus* (n = 23)	*H. aratta* sp. n. (n = 15)	*H. enischnochela* (n = 26)	*H. jiroftensis* (n = 8)	*H. lepturus* (n = 11)	*H. persicus* (n = 5)	ANOVA	HSD
MT1L	4.54 ± 0.85	5.67 ± 0.72	7.47 ± 1.46	7.79 ± 0.85	6.80 ± 0.51	5.67 ± 0.02	F =15.01 $p < e^{-8}$	ac<<<en=ji=le, ar<<en=ji
MT2L	4.96 ± 0.88	6.39 ± 0.70	8.30 ± 1.67	8.45 ± 0.86	7.57 ± 0.40	6.28 ± 0.39	F =15.52 $p < e^{-8}$	ac<<<en=ji=le, ar<<en, ar<ji
MT3L	5.19 ± 0.93	6.70 ± 0.88	8.68 ± 1.69	8.83 ± 1.11	7.69 ± 0.43	6.67 ± 0.16	F =14.92 $p < e^{-7}$	ac<<<en=ji=le, ar<<en, ar<ji
MT4L	5.69 ± 1.03	7.01 ± 0.81	8.96 ± 1.68	9.25 ± 1.04	8.03 ± 0.34	6.70 ± 0.33	F =13.79 $p < e^{-7}$	ac<<<en=ji, ac<<pe, ar<<en, ar<ji
MT5L	6.22 ± 1.04	7.94 ± 0.92	9.26 ± 1.54	10.02 ± 1.18	7.77 ± 1.08	7.78 ± 0.57	F =12.05 $p < e^{-6}$	ac<<<en=ji, ac<ar, le=ar<ji
TL	4.77 ± 0.79	5.60 ± 0.88	6.20 ± 1.00	6.78 ± 0.78	6.07 ± 0.69	5.49 ± 0.18	F =6.17 $p < e^{-2}$	ac<<<en=ji, ac<le
BTL	47.99 ± 8.35	59.71 ± 5.74	72.80 ± 11.91	78.57 ± 9.08	65.88 ± 3.90	61.68 ± 4.03	F =14.93 $p < e^{-7}$	ac<<<en=ji, ac<<pe, ar<en, ar<<ji
FL/W	2.77 ± 0.18	2.81 ± 0.64	3.29 ± 0.28	2.93 ± 0.29	2.58 ± 0.17	2.92 ± 0.04	F =6.19 $p < e^{-2}$	ac<<en, le<<<en, ar<en
PL/W	2.44 ± 0.09	2.50 ± 0.19	2.63 ± 0.16	2.72 ± 0.16	2.48 ± 0.17	2.56 ± 0.19	F =4.11 $p < e^{-2}$	ac<en, ac<<ji
ML/W	2.70 ± 0.21	1.66 ± 0.35	3.01 ± 0.62	1.41 ± 0.09	1.38 ± 0.06	1.64 ± 0.11	F =31.88 $p < e^{-13}$	ji=le=ar<<<ac, pe<ac, ji=le=pe=ar<<<en
MSL/MSW	2.80 ± 0.22	2.86 ± 0.29	2.72 ± 0.34	3.52 ± 0.55	2.77 ± 0.49	3.35 ± 0.22	F =5.40 $p < e^{-2}$	ac=le<<ji, en<<<ji, ar<ji
MT1L/W	3.14 ± 0.27	3.30 ± 0.28	3.74 ± 0.56	3.93 ± 0.48	3.73 ± 0.35	3.69 ± 0.40	F =4.87 $p < e^{-2}$	ac<<en=ji
MT2L/W	3.95 ± 0.41	4.30 ± 0.42	4.89 ± 0.84	5.04 ± 0.61	4.60 ± 0.24	4.47 ± 0.22	F =4.78 $p < e^{-1}$	ac<<en=ji
MT3L/W	4.38 ± 0.36	4.70 ± 0.56	5.61 ± 0.97	5.72 ± 0.86	5.05 ± 0.38	5.19 ± 0.33	F =5.90 $p < e^{-2}$	ac<<en, ac<<ji, ar<n
MT4L/W	5.14 ± 0.43	5.80 ± 0.60	5.94 ± 0.97	6.67 ± 0.95	6.14 ± 0.80	6.38 ± 0.11	F =3.96 $p < e^{-1}$	ac<<ji
MT5L/W	5.52 ± 0.64	6.27 ± 0.84	5.91 ± 0.82	6.95 ± 0.95	5.35 ± 0.79	6.82 ± 0.59	F =4.26 $p < e^{-1}$	ac<<ji

Table S3. Statistics (mean ± standard deviation) for morphometric characters with sexual dimorphism in females of six Iranian species of *Hemiscorpius* Peters, 1861 (based on ANOVA and Tukey posthoc HSD tests (<, << and <<< indicate *p*-values less than 0.05, 0.01, and 0.001, respectively). Abbreviations: ac = *H. acanthocercus*; ar = *H. aratta* sp. n.; en = *H. enischnochela*; ji = *H. jiroftensis*; le = *H. lepturus*; pe = *H. persicus*.

Character	*H. acanthocercus* (n = 23)	*H. aratta* sp. n. (n = 15)	*H. enischnochela* (n = 26)	*H. jiroftensis* (n = 8)	*H. lepturus* (n = 11)	*H. persicus* (n = 5)	ANOVA	HSD
CL	5.58 ± 0.79	5.81 ± 0.37	7.20 ± 1.57	7.56 ± 1.57	6.67 ± 0.33	6.04 ± 0.91	F =3.40 $p < e^{-1}$	ac<en
FW	1.78 ± 0.20	1.99 ± 0.16	2.13 ± 0.44	2.64 ± 0.26	2.30 ± 0.09	1.96 ± 0.33	F =4.19 $p < e^{-2}$	ac<<ji

Table S4. Areas (km^2) of habitat suitable for eight Iranian species of *Hemiscorpius* Peters, 1861.

Variables	*H. acanthocercus*	*H. aratta* sp. n.	*H. enischnochela*	*H. jiroftensis*	*H. kashkayi*	*H. lepturus*	*H. persicus*	*H. shahii*
High suitability	28,609 (1.7%)	118,397 (7%)	18,235 (1%)	199,341 (12%)	7,070 (0.4%)	58,846 (3.5%)	111,212 (6.7%)	25,265 (1.5%)
Moderate suitability	20,532 (1.2%)	68,763 (4.5%)	25,702 (1.5%)	31,228 (2%)	2,692 (0.2%)	15,128 (1%)	1,072 (0.1%)	3,131 (0.2%)
Low suitability	6,158 (0.4%)	172,564 (10.5%)	8,922 (0.7%)	380,427 (23%)	4,902 (0.4%)	25,013 (1.5%)	180,851 (11%)	31,315 (1.9%)
Unsuitable	1,592,896 (96.7%)	1,288,471 (78%)	1,595,335 (96.8%)	1,037,197 (63%)	1,633,529 (99%)	1,549,207 (94%)	1,355,059 (82.2%)	1,588,484 (96.4%)
